# Current and Novel Inhibitors of HIV Protease

**DOI:** 10.3390/v1031209

**Published:** 2009-12-11

**Authors:** Jana Pokorná, Ladislav Machala, Pavlína Řezáčová, Jan Konvalinka

**Affiliations:** 1 Institute of Organic Chemistry and Biochemistry, Academy of Sciences of the Czech Republic, Gilead Sciences and IOCB Research Center, Flemingovo n. 2, 166 10, Prague 6, Czech Republic; E-Mails: jana.pokorna@uochb.cas.cz (J.P.); pavlina.rezacova@uochb.cas.cz (P.R.); 2 AIDS Center, Department of Infectious Diseases, University Hospital Bulovka, Budínova 2, 120 00, Prague 8, Czech Republic; E-Mail: ladimachala@centrum.cz; 3 Department of Infectious Diseases, Third Faculty of Medicine, Charles University in Prague, Prague, Czech Republic; 4 Institute of Molecular Genetics, Academy of Sciences of the Czech Republic, Flemingovo n. 2, 166 10, Prague 6, Czech Republic; 5 Department of Biochemistry, Faculty of Science, Charles University in Prague, Hlavova 8, 128 43, Prague 2, Czech Republic

**Keywords:** HIV protease, protease inhibitors, HAART, resistance development, pharmacokinetic boosting, protease dimerization, alternative inhibitors

## Abstract

The design, development and clinical success of HIV protease inhibitors represent one of the most remarkable achievements of molecular medicine. This review describes all nine currently available FDA-approved protease inhibitors, discusses their pharmacokinetic properties, off-target activities, side-effects, and resistance profiles. The compounds in the various stages of clinical development are also introduced, as well as alternative approaches, aiming at other functional domains of HIV PR. The potential of these novel compounds to open new way to the rational drug design of human viruses is critically assessed.

## 1. Introduction

**Figure 1.**
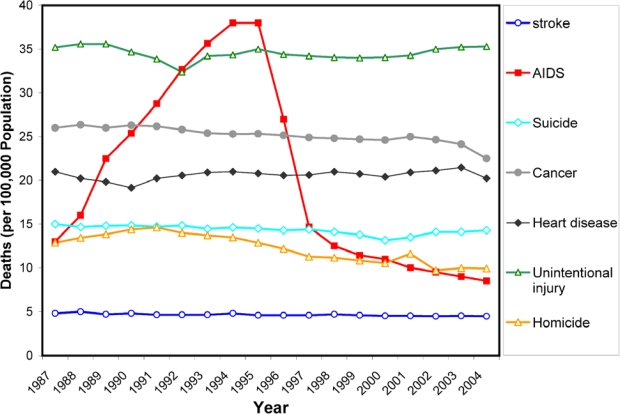
Trends in annual rates of death due to 7 leading causes among persons 25–44 years old in the United States during period 1987–2004. Dramatic decrease in the rate of death due to AIDS coincides with the introduction of HIV protease inhibitors (source: National Vital Statistics, Centers for Disease Control and Prevention, Atlanta).

The aspartic protease of human immunodeficiency virus (HIV PR) is responsible for the cleavage of the viral Gag and Gag-Pol polyprotein precursors into mature, functional viral enzymes and structural proteins. This process, called viral maturation, which leads to the final morphological rearrangements, is indispensable for production of infectious viral particles [1]. If HIV-PR is inhibited, the nascent virions cannot go on to attack other cells and the spreading of HIV is therefore stopped. The introduction of HIV protease inhibitors (PIs) in 1995 and the application of highly active anti-retroviral therapy (HAART), *i.e.*, combination of PI with other antiretrovirals, mainly inhibitors of the HIV reverse transcriptase, resulted in a vastly decreased mortality (Figure 1) and a prolonged life expectancy of HIV-positive patients. The success story of the therapeutic use of HIV protease inhibitors is not only a remarkable achievement of modern molecular medicine, but it also represents a unique showcase for the power and limitations of a structure-based drug design in general.

Although the success of HIV PIs has been remarkable and there are not fewer than nine of these compounds currently approved by the FDA as antiviral agents, for several reasons both the academia as well as the industry need to continue in their effort to develop novel, more potent compounds.

**Figure 2.**
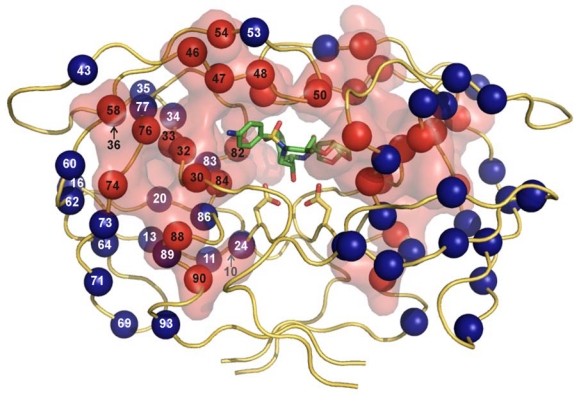
The three-dimensional crystal structure of HIV PR dimer depicting mutations associated with resistance to clinically used protease inhibitors [7]. Mutated residues are represented with their Cα atoms (spheres) and colored in the shades of red and blue for major and minor mutations, respectively. For major mutations, the semi-transparent solvent accessible surface is also shown in red. Active site aspartates and PI darunavir bound to the active site are represented in stick models. The figure was generated using the structure of highly mutated patient derived HIV-1 PR (PDB code 3GGU [8]) and program PyMol [9].

First, there is a problem of antiviral drug resistance. The high mutation rate caused by the lack of proofreading activity of the viral reverse transcriptase, the dynamic viral replication in HIV-positive individuals, together with potential dual infection and insufficient effect of drugs lead to rapid selection of viral species resistant to the currently used inhibitors. The pattern of mutations associated with the viral resistance is extremely complex (Figure 2, Table 1). The mutations are selected not only in the protease substrate binding cleft in the direct proximity of an inhibitor, but also outside the active site of the enzyme. Besides the common mechanism of amino acid substitution, insertions in the PR have also been observed to be selected during antiretroviral therapy [2,3]. Furthermore, HIV occasionally rescues its fitness under the selection pressure of PIs by changing the protease substrate, *i.e.*, the polyprotein cleavage sites [4,5]. Some of these *gag* mutations have been shown to confer the viral resistance even without the corresponding changes in the PR [6]. The mechanism of resistance development and the structural aspects of the interaction of PIs with the active cleft of mutated HIV PR species are discussed in other reviews in this volume.

Secondly, the clinical use of PIs is further affected by their high price and by problems of tolerability, toxicity, and adherence. Before the introduction of the first PIs into the clinical practice, optimistic expectations prevailed that the toxicity of this class of virostatics will be low because of the absence of enzymes similar to HIV protease in the human body. Unfortunately, the reality was different, and soon it turned out that compounds can interact with other molecules, particularly in the lipid metabolism and trafficking pathways. Consequently, the side effects of PIs are very frequent and often so serious that the drug toxicity may sometimes represent even a greater risk for patients than the HIV infection itself [10–12]. The presence of side effects together with the pill burden negatively influence the patient’s adherence and hence contribute to the evolution of resistance.

Taken together, inspite of the indisputeble success of the HAART and benefit to patients, new approaches to the antiviral treatment, including novel HIV PIs, are highly desirable to achieve better control of HIV infection while maintaining acceptable quality of patient’s life. Novel PIs should be developed with broad specificity against PI-resistant HIV mutants, better pharmacokinetic properties, lower toxicity, and simple dosage.

In the following contribution, we will briefly review the PIs that are currently used clinically, and we will also mention some other compounds that entered various stages of clinical testing. Subsequently, we will provide an overview of selected experimental HIV PIs targeting the enzyme active site as well as other functionally important regions, namely the protease dimerization and flap domains.

## 2. Inhibitors currently used in clinical practice: structure, antiviral activity, side-effects, and pharmaceutical boosting

Since the identification of HIV as an etiological entity of AIDS, more than twenty different drugs have been introduced into the clinical practice. Such drugs inhibit specific steps in viral lifecycle and thus the replication of the virus. In fact, more virostatics are available for HIV/AIDS than for all other viruses combined. This exciting endeavor has been extensively reviewed [13–21]. In developed countries, HAART considerably changed the course of HIV infection to a controllable and treatable long-term disease.

Nine protease inhibitors are currently on the market (see Table 2). They are competitive inhibitors of HIV PR and all but one are peptidomimetics of the polyprotein cleavage sites. The first true non-peptidic inhibitor is tipranavir, which was approved in 2005. The currently available PIs are also active against the HIV-2 protease, but with some exceptions – e.g., lopinavir is less effective in the therapy of HIV-2 infection [22]. As mentioned above, important characteristics of any drug that are very relevant for its clinical application are toxicity and undesired side effects. The pathogenesis of some side effects has already been satisfactorily explained (e.g., indinavir nephrolithiasis occasionally caused by indinavir), but pathogenetic mechanisms of numerous side effects (e.g., lipodystrophy syndrome, insulin resistance, inhibition of glucose uptake or disturbances of bone metabolism) is still not entirely elucidated [23–26], reviewed in [21] .

All PIs in current clinical practice have been approved only for oral administration, but their absorption in the gastrointestinal tract and diffusion through anatomical barriers is usually poor to moderate. This effect is partly due to their extensive binding to plasma proteins (90–99%), partly due to the binding to P-glycoprotein and consequent eflux of the drug [27]. These factors make their effective concentration low, particularly in sanctuary sites such as the central nervous system [28] or the genital tract [29,30]. In order to suppress viral infection, drug levels above suitable inhibitory concentration must be achieved and maintained in the bloodstream as well as in the virus sanctuary sites. Lower drug concentrations in the target sites might lead to ongoing viral replication and enable the emergence of drug-resistant variants of the virus. It is therefore very important that many pharmacokinetic limitations of PIs can be partially overcome by co-administration with low doses of a pharmacokinetic enhancer, or “booster”, a potent inhibitor of cytochrome P450 such as ritonavir (see below). Ritonavir-boosted PI therapy leads to simplified treatment regimens, reduced pill burden, and, consequently, better patient adherence. This, in turn, helps to prevent the emergence of antiviral resistance. At present, all currently marketed PIs, with the exception of nelfinavir, are prescribed with a low dose (100 mg per day) of ritonavir as a booster. Although the mechanism of P450 inhibition by ritonavir (RTV), originally developed as HIV PR inhibitor, is not fully understood, rational design of cytochrome P450 inhibitors has become a very active research field in current pharmaceutical science [31] (for recent review see [32,33]). Several promising compounds are currently in various stages of clinical testing (e.g., Gilead Sciences’ GS-9350, SPI-452 from Sequoia Pharmaceuticals, TMC-41629 from Tibotec, PF-03716539 from Pfizer, as well as some others, reviewed in [21,32].

On the other hand, any co-administration of these “boosters” with drugs metabolized by cytochrome P450 isoenzyme 3A4 may be problematic and lead to severe side-effects. There have been reports on serious and even life-threatening events following co-administration of PIs with hypolipidemics (HMG-coA reductase inhibitors), antihistamines, or ergot alkaloids. Numerous other drugs, such as, e.g., sildenafil, corticosteroids, or psychotropic agents [34], also need to be prescribed with caution to patients on HAART therapy involving pharmacokinetic boosters.

### 2.1. The first generation HIV protease inhibitors

**Figure 3.**
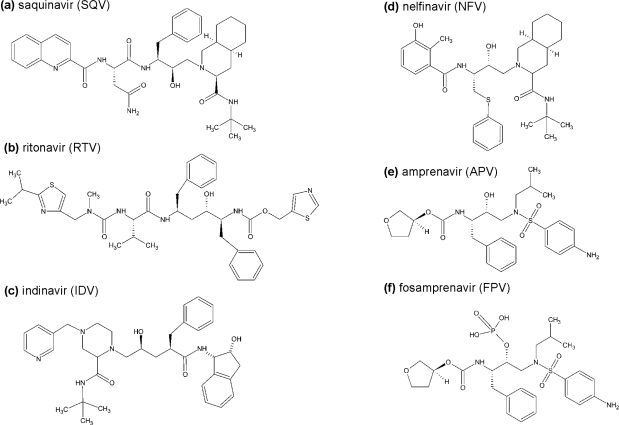
Chemical structures of the first generation HIV protease inhibitors.

The design of the first protease inhibitor **saquinavir** (SQV, Ro-31-8959, marketed by Hoffman-La Roche), which was approved by the FDA in December 1995 under the commercial name Invirase®, was based on rather unusual specificity of HIV protease for cleavage of the Phe-Pro and Tyr-Pro bond (Figure 3a). The scissile bonds were replaced by a hydroxyethylamine isostere [35]. Despite its high *in vitro* antiviral activity, the clinical efficacy of this drug was significantly reduced due to the insufficient bioavailability (less than 4%) and intense hepatic metabolism. In order to improve the bioavailability of SQV, a novel formulation of the drug in soft capsules was developed and marketed under the name of Fortovase® (Hoffman-La Roche) [36]. Subsequently, SQV was co-formulated with with ritonavir as a booster. As of February 15, 2006, the sales of Fortovase were discontinued in the United States because of poor drug tolerance; Invirase boosted by ritonavir remained the only drug formulation of SQV currently available on the market.

Typical primary resistance-conferring mutations selected under the pressure of SQV are G48V or L90M. The most common adverse reactions reported in patients receiving SQV were gastrointestinal disturbances, such as diarrhea, nausea, or abdominal discomfort [37].

**Ritonavir** (RTV, ABT-538) was designed by Abbot laboratories and gained FDA approval in 1996 under the name Norvir® [38]. The drug design process originally concentrated on C-2 symmetric molecules. However, the structures of the inhibitor/enzymes complexes proved that even the most active C-2 symmetric compound loses symmetry when bound to the active site of the enzyme. Therefore, the final structure of the inhibitor is asymmetrical (Figure 3b), which also enables improvement of bioavailability by different modifications of the end groups. Moreover, C-2 symmetric compounds would be more sensitive to PR resistance development since every mutation in PR would affect the inhibitor binding twice.

RTV inhibits both HIV-1 and HIV-2 proteases. *In vivo,* the major observed mutations decreasing the susceptibility of enzyme to RTV are I84V and V82A. Ritonavir was originally approved as an antiretroviral drug, but due to frequent occurrence of side effects (such as gastrointestinal symptoms like nausea, abdomenalgia and diarrhea) and high cross-resistance with other PIs, its use as a HIV-protease inhibitor was gradually abandoned. Unexpectedly, RTV has proven to be a potent inhibitor of cytochrome P-450 3A4, the microsomal enzyme responsible for the bulk of metabolism of other PIs (as well as other xenobiotics, see above). Therefore, ritonavir is currently used almost exclusively as a pharmacokinetic boosting agent which increases the plasma concentration of other PIs and thus prolongs their therapeutic effects [39].

**Indinavir** (IDV, MK-639, L-735,524), manufactured by Merck & Co, was approved in 1996 under the trade name Crixivan® [40]. Design of this compound possessing a hydroxyaminopentanamide transition state isostere was based on the framework of previously developed renin inhibitors. Physical properties, *i.e.*, solubility and lipophilicity, of optimized molecules were further improved using molecular modeling and X-ray crystal structure analysis of the inhibitor/enzyme complexes (Figure 3c). Although indinavir used to be among the most widely clinically used first-generation PIs, at present it has been almost completely abandoned due to toxicity and frequent side effects. Nephrolithiasis is a typical side effect of IDV caused by disturbances of the drug solubility in urine. The compound is more soluble in acidic urine due to the presence of the basic polar groups. With the increase of the actual pH (>5.5), IDV tends to crystallize in kidneys [41]. Another typical side effect of IDV is the lipodystrophy syndrome, characterized by maldistribution of body fat (accumulation in abdominal area – “crix belly” and “buffalo hump”) [42].

The first protease inhibitor which could be used by adults as well as by children was **nelfinavir** (NFV, AG-1343), approved in 1997 and marketed as Viracept®. Developed by Agouron Pharmaceuticals by iterative protein co-crystal structure analysis, it was the first PI which structure has been optimized by Monte Carlo simulations [43] (Figure 3d). In more than a half of the patients, resistance conferring mutation D30N is selected, often accompanied by N88D. The other possible pathway leading to NFV resistance involves the selection of L90M mutation. Interestingly, these two pathways seem to be mutually exclusive [44]. The therapeutic use of NFV is currently limited because of the significantly lower efficacy compared to the recently introduced second generation PIs. The major adverse effect of NFV is gastrointestinal toxicity characterized by diarrhea, often of a severe grade. Other common side effects are metabolic disturbances, such as lipodystrophy and hyperlipidemia [45].

**Amprenavir** (APV, 141W94 or VX-478) from Vertex Pharmaceuticals and GlaxoSmithKline has been marketed under the name Agenerase® since 1999 [46]. Its central part is derived from SQV, but the P2 end group is replaced by tetrahydrofurancarbamate and the P1′-P2′ moiety by a sulfonamide derivative (Figure 3e). This structure has some advantages over SQV such as fewer chiral centers, easier synthesis, and higher oral bioavailability. Mutations which result in a decrease of susceptibility to Agenerase® occur mainly in positions M46I/L, I47V, I50L, I54L/V, and I84V. Mutations conferring resistance to APV were also observed in the p1/p6 of the Gag substrate cleavage site, at positions L449 and P453 [47]. The adverse events of APV are mainly gastrointestinal, such as loose stools and abdominal pain; skin rash is also relatively common. APV administration could also lead to hypertriglyceridemia and hypercholesterolemia [48–50].

In order to further improve the safety, pharmacokinetics, and therapeutic potency of amprenavir, **fosamprenavir** (FPV,fAPV, GW 433908, VX-175), a hydrophilic phosphate ester prodrug of amprenavir, was developed by Vertex Laboratories and GlaxoSmithKline [51] (Figure 3f), and has been marketed under the name Lexiva® since 2003. *In vivo*, FPV is quickly converted to APV by host phosphatases during absorption in the gut. Several mutations, namely V32I, I47V/A, I50V, I54L/M, L76V and I84 V/A, are relative contraindications to the use of FPV. The side effects of FPV are comparable to those of APV [52].

### 2.2. The second generation HIV protease inhibitors

The second generation inhibitors were designed to inhibit HIV PR species resistant to the inhibitors of the first generation and to introduce well tolerated drugs with minimized side effects and simple once-daily dosing, which may improve patients’ adherence to the treatment.

**Figure 4.**
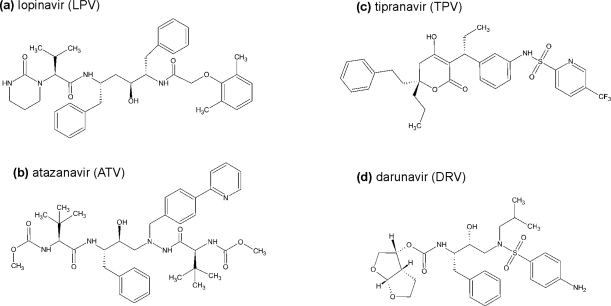
Chemical structures of the second generation HIV protease inhibitors.

**Lopinavir** (LPV, ABT-378) is the second generation inhibitor most widely used in drug-naïve patients. Developed by Abbott, it has been marketed in a co/formulation with ritonavir under the name Kaletra® since 2000. LPV was designed to inhibit resistant PR species that contain the common mutation V82A. The molecular design of LPV was based on the structure of ritonavir and began by eliminating the P3 isopropylthiazolyl group of RTV interacting with the V82 residue of the wild-type PR. Further replacement of the thiazolylmethoxycarbonyl moiety in the P2′ with dimethylphenoxyacetyl group yielded LPV (Figure 4a). The core P1-P1′ positions are thus occupied by the same hydroxyethylene peptidomimetic as in RTV [53].

At present, Kaletra® is the first choice PI for an initial antiretroviral regimen. The development of resistance is associated with accumulation of mutations occurring at nine to eleven positions (“lopinavir mutation score”) [54]. Furthermore, a single mutation I47A, contributing to the reduced sensitivity of HIV-1 [55–57] and also HIV-2 [58] protease to the LPV, has been reported [59]. Nevertheless, this mutation compromises the replication capacity of the corresponding virus ([58] and the citations therein), thus explaining its low prevalence in the HIV-positive patients. The side effects of LPV are similar to those of other PIs. The major problems are gastrointestinal events, such as abdominal pain, gaseous symptoms or diarrhea, and metabolic disturbances like hypercholesterolemia and hypertriglyceridemia [60].

The structure-based design of **atazanavir** (ATV, CGP 73547, BMS-232632) was part of a complex exploration of azadipeptide analogues [61] (Figure 4b). Originally developed by Ciba-Geigy and marketed since 2003 under the trade name Reyataz® by Bristol-Myers Squibb, atazanavir was the first PI that was compatible with once-daily dosing. The compound shows the least binding to the serum proteins, minimal effect on insulin-stimulated glucose uptake and appeared to be less likely to cause lipodystrophy and high level of cholesterol as side effects. The main mutations contributing to the resistance are I50L, I84V, and N88S. In patients treated with ATV, a prominent indirect hyperbilirubinaemia frequently occurs, often leading to clinically apparent jaundice. This condition is, indeed, not dangerous, but may have an undesirable cosmetic effect, which may discourage the patient from the therapy [62].

A unique compound was designed at Pharmacia & Upjohn and later introduced by Boehringer-Ingelheim. **Tipranavir** (TPV, PNU-140690), approved under the name Aptivus® in 2005, is a non-peptidic inhibitor, belonging to the 4-hydroxy-5,6-dihydro-2-pyrone sulfonamides [63] (Figure 4c). Despite its structural differences from the peptidic inhibitors, its interactions with HIV PR are similar in many respects, as shown by its crystal structure [64]. Nevertheless, TPV shows considerable activity against numerous PI-resistant HIV strains [65]. As many as 16–20 mutations in the protease gene are needed for significant decrease of the HIV PR susceptibility [66]. In addition to the gastrointestinal and metabolic disturbances that are common for the whole class, the main limitation of the drug is its hepatotoxicity [67]. Occasionally, intracranial hemorrhaging has also been reported [68]. Since it is a CYP450 3A4 inducer, TPV requires a double dosage of RTV as a pharmacokinetic booster [69].

The most recently (2006) approved nonpeptidic HIV protease inhibitor is **darunavir** (DRV, TMC-114, UIC-94017, trade name Prezista®) [70]. Developed by Tibotec BVBA, formerly Tibotec-Virgo NV, it is a structural homolog of TMC-126 (UIC-94003). Both compounds are chemically similar to APV, but differ by the presence of a bis-tetrahydrofuranyl moiety, (Figure 4d**)**, which forms a crucial hydrogen bonding interactions with the main chain of Asp29 and Asp30 in the PR. These polar interactions with the backbone atoms in the PR active site enable DRV to mimic the conserved hydrogen bonds of the natural substrates [71]. Its broad specificity against mutated, highly resistant PR species could be interpreted by its ability to fit to the proposed ”substrate envelope” within the active site [72,73]. Recently, ultra-high resolution crystal structures revealed that DRV binds not only to the active-site cavity, but can also occupy a surface pocket formed by one of the flaps (flexible loops covering the enzyme active site) [74]. Enzyme kinetic experiments further confirmed the notion that both DRV and APV have a second binding site outsite the active cleft [75]. An assay based on fluorescent resonance energy transfer suggests that DRV has a dual mechanism of inhibition, as it might also be a potential PR dimerization inhibitor [76].

Even though DRV is chemically related to APV, it binds nearly 100 times more tightly than APV and 1,000 times more tightly than SQV, IDV, RTV and NFV to the wild-type HIV-1 protease [77,78]. In clinical studies with treatment experienced and naïve individuals, patients developing darunavir resistance tend to have a high number of PI resistance-associated mutations. Thus, the genetic barrier to the development of resistance, a measure of the difficulty for the virus to escape from the selective pressure of the drug by developing mutations, is very high for DRV. The substitutions suggested to be critical for the development of DRV resistance were V11I, V32I, L33F, I47V, I50V, I54L/M, G73S, T74P, L76V, I84V, and L89V [79–81]. Grantz Saskova *et al.* have recently characterized a virus strain isolated from HIV-positive patient under prolonged treatment by DRV that contained as many as 22 mutations in the PR region and exhibited pronounced DRV resistance [8].

The high genetic barrier to the development of resistance, its better clinical efficacy against multidrug-resistant HIV variants together with favorable tolerability, safety, and once-daily regime, make DRV one of the preferred therapeutic option both for drug-naïve and for highly treatment-experienced patients. DRV is generally well tolerated, but the main adverse effects are common with the other members of the class, such as gastrointestinal symptoms and lipid abnormalities [82].

## 3. Inhibitors of HIV protease in the pipeline

Some other interesting compounds were recently or are now in various stages of clinical trials. The often convoluted history behind the development of individual compounds documents the complex requirements for the activity, resistance profile, toxicity, pharmacokinetics, and drug interactions, as well as fierce competition in the field.

**Figure 5.**
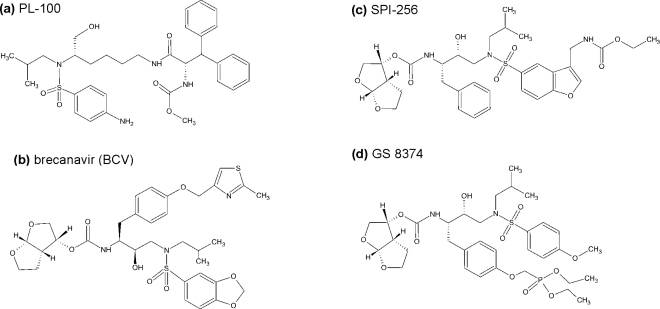
Chemical structures of inhibitors HIV protease in the pipeline.

Compound **PL-100,** a lysine-sulfonamide inhibitor with high genetic barrier to the development of resistance and with a favorable cross-resistance profile has been developed by Ambrilia Biopharma Inc. (formerly Procyon) and licenced by Merck Co. as Mk 8122 [83]. The compound has been identified in a compound library based on l-lysine scaffold (Figure 5a). It is highly active against HIV PR and numerous resistant mutants and shows high genetic barrier towards the development of resistant virus strains. Moreover, it also possesses inhibitory activity against CYP450 3A4/5, which brings about the potential of using this compound in a once-daily, un-boosted regime. Crystal structure of a complex of HIV PR with a close analog of PL-100, lysine-sulfonamide 8, shows direct H-bond interactions with the flap, displacing the conserved flap water molecule [84]. Despite these favorable features, Merck announced in the summer of 2008 that it will put “development of PL-100 on hold and will concentrate on other PL-100 prodrugs, formulation options, and back-up compounds”.

**Brecanavir** (BCV, GW 640385), developed in a collaborative effort of GlaxoSmithKline and Vertex, is a tyrosyl-based arylsulfonamide protease inhibitor with relatively low binding to the plasma proteins and high affinity against a variety of PI-resistant viral species (Figure 5b). It was reported to exhibit higher *in vitro* potency than APV, IND, LPV, ATV, TPV, and DRV, which makes it the most potent and broadly active antiviral agent among the PIs tested *in vitro* [85]. However, in December 2006, GSK announced that it discontinued development of BCV, which was then in phase II clinical development, “due to insurmountable issues regarding formulation”.

A structure-based approach at Sequoia Pharmaceuticals, Inc. involved identification of a substructure of conserved regions in the PR active site and the design of compounds that would make optimal interactions with such a conserved substructure. The aim was to design compounds retaining high potency against a variety of PI-resistant HIV strains. This effort lead to discovery of **SPI-256** (Figure 5c). This compound, currently in phase I clinical evaluation, is highly active against wild-type and multidrug-resistant HIV PRs, with inhibition constants in the picomolar range. It shows high genetic barrier, and exhibits a better resistance profile than any of the current FDA-approved compounds when analyzed using PhenoSense assay [86].

**GS 8374,** developed at Gilead Sciences [87], is based on the darunavir scaffold (more specifically, on TMC 126) with covalent attachment of a phosphonic acid moiety (Figure 5d). The phosphonate compound exhibits high affinity to HIV-1 protease, considerable antiretroviral activity, and a more favorable cross-resistance profile against clinically relevant PI-resistant HIV-1 strains. Its co-crystal structure suggests that the phosphonate group, exposed to the solvent, brings about a favorable change in the inhibitor binding entropy after the interaction with mutant enzymes via “anchoring” of the inhibitor molecule to the bulk solvent. GS 8374 showed a resistance profile superior to LPV, ATV, and DRV when assayed against a panel of highly resistant mutant viruses [88].

## 4. Other non-peptidic HIV protease active site inhibitors

Generally, the design of novel HIV PIs includes efforts to minimize the inhibitor molecular weight, maximize its interactions with the backbone of the PR binding cleft while maintaining flexibility for better fit to the variable binding clefts of PR resistant species [89]. These general principles are being used by scientists from industry and academia in their efforts to design the next generation of HIV PIs.

The variability of structural motifs used by rational design or selected by library or combinatorial screening is enormous, and it is beyond the scope of this review to attempt to cover all promising compounds reported up to date. Below we will only mention several interesting recent examples, thus exemplifying the variability of approaches and of the resulting chemical structures. Even though it is questionable whether any of these compounds will ever enter the market, they still represent important contributions to the armamentarium of modern medicinal chemistry.

Favourable properties of darunavir lead to the development of several follow-up compounds, exemplified by **GRL** series by Ghosh *et al.* The P2 bis-tetrahydrofuranyl residue of darunavir was replaced by its hexahydrocyclopentafuranyl (GRL -06579A) and P2-P1′ positions of the parent structure were modified by pyrrolidinone and oxazolidinone derivatives (GRL 02031), retaining high antiviral activity and favourable resistance profile [90,91].

**Figure 6.**
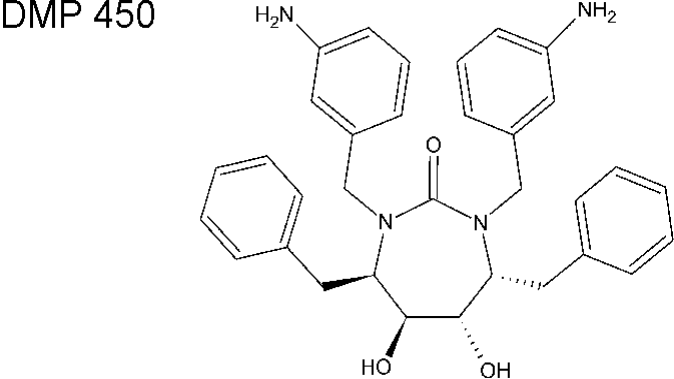
Chemical structure of DMP450 (*Mozenavir (DuPont)*.

A very attractive class of compounds, cyclic ureas, was introduced in 1994 by Lam *et al.* [92]. These non-peptidic compounds were designed to include a mimic of the water molecule in the flap-proximal part of the enzyme active site. Such a water molecule was shown to interact with the main chain atoms of the closed flaps for the substrate and almost all peptidic inhibitors. Several cyclic compounds were prepared, with analogues including a seven-membered ring containing compounds **DMP323** [93] and **DMP450** (Figure 6) [94]. Hallberg group in Uppsala have used carbohydrates (mannitol) as chiral precursors for the synthesis of several cyclic and *C*2-symmetric urea and sulfamide inhibitors [95,96]. Although there is currently no information about any cyclic urea-based compounds entering clinical trials, the 7-membered ring of cyclic urea is still being used as an useful scaffold for further PI design.

**Figure 7.**
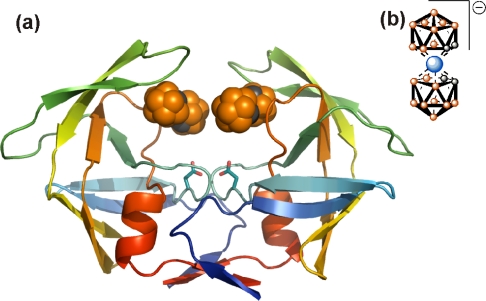
Crystal structure of metallacarborane inhibitor bound to HIV PR. **(a)** Two metallacarborane clusters bind to the flap-proximal part of the active site. The HIV PR is represented by a ribbon diagram and colored by rainbow from blue to red (N- to C-termini), the atoms of the metallacarborane cluster are represented by spheres and colored orange for boron atoms, gray for carbon atoms, and blue for cobalt. The structural formula is depicted in **(b).** Hydrogens are omitted for clarity.

In order to enlarge the chemical space available for the design of novel anti HIV molecules, several groups used unusual chemistry for the identification of HIV PIs. Surprisingly, even inorganic compounds, **Nb-containing polyoxometalates**, specifically inhibit HIV PR with submicromolar potency in tissue cultures [97]. In this case, the inhibitors were shown to be non-competitive and a model suggested binding to the cationic pocket on the outer surface of the flaps (see below). Clearly, the active site of HIV PR could also be targeted by compounds with unexpected chemistry. The HIV PR binding cleft was shown to accommodate **C_60_ fullerenes**, and some fullerene derivatives are indeed weak inhibitors of HIV PR [98–100]. In a search for other unconventional chemical structures that would fit into the PR binding cleft, and possess favorable pharmacologic properties, Cigler *et al.* [101] recently identified a group of inorganic compounds, **icosahedral carboranes**, as promising scaffolds for PIs. Boron-containing compounds and carboranes specifically have been already utilized in medicinal chemistry in boron neutron capture therapy and in radioimaging. Such compounds are also used as stable hydrophobic pharmacophores, usually replacing bulky aromatic amino acid side chains [102,103]. Bis(dicarbollides) or metallacarboranes that consist of two carborane cages sandwiching the central metal atom, were shown to be specific, stable and rather potent inhibitors of HIV PR [101,104,105]. A crystal structure revealed binding of two bis(dicarbollide) clusters to hydrophobic pockets in the flap-proximal region of the HIV PR active site, “above” the site for conventional active-site inhibitors (Figure 7). This unusual binding mode might explain the broad inhibition activity of metallacarboranes against highly PI-resistant HIV PR variants [104].

In order to rationalize the design of new generation PIs, Schiffer and coworkers introduced an interesting approach (recently reviewed in [106]). They proposed that a structural space within the HIV PR binding cleft, defined by a consensus volume occupied by the natural substrates of the enzyme, should represent a spatial constraint for the inhibitor design. If the inhibitor binds within this “substrate envelope”, which was identified by a series of detailed structural analyses of enzyme-substrate complexes [107], then its activity should not be significantly compromised by mutations in the PR binding cleft. Any mutation within the “substrate envelope” would necessarily lead to a substrate binding defect. This approach has been tested on a series of compounds based on amprenavir structure and designed to fit within the substrate envelope. Some of these compounds exhibited picomolar inhibitory constants against a panel of multi-resistant HIV PR variants [108].

## 5. Alternative HIV protease inhibitors targeting functional domains outside the enzyme active site

Several HIV PR regions have been identified that seem to be conserved in all examined HIV sequences derived from treatment-naïve patients. They include residues 1–9 and 94–99 (N- and C- termini), 21–32 (active site core), 47–56 (flap region) and 78–88 (substrate-binding region) [109]. Although the resistant mutations could clearly evolve even in these conserved regions (especially in the flap and substrate-binding region), it is tempting to suggest that compounds binding conservative domains of the enzyme outside the active site might be “resistance-repellent”. Moreover, inhibitors targeted to the domains outside the active cleft might show a synergistic effect to the conventional active-site targeted compounds. Finally, blocking an earlier event in the maturation pathway of the virus, such as HIV PR dimerization by binding Gag-Pol polyprotein prior viral maturation, might be an attractive approach for antiviral therapy. The alternative inhibitor designs have not, as yet, yielded any successful drugs. However, the approaches and techniques developed for their creation could be proven useful in the future, or in design of inhibitors of other targets.

### 5.1. HIV PR dimerization inhibitors

**Figure 8.**
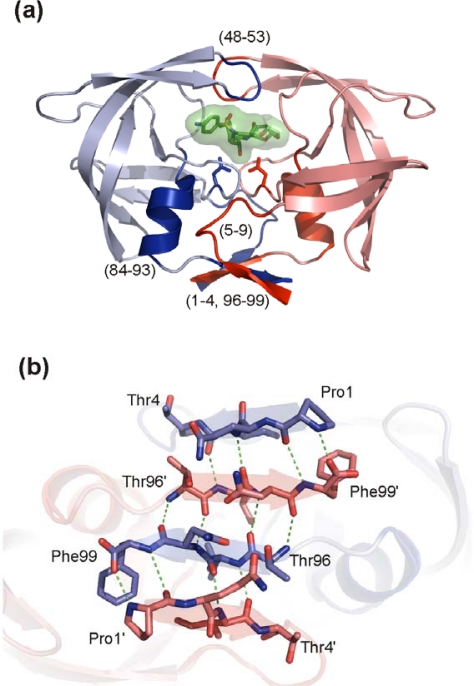
HIV PR dimerization interface. **(a)** The overall structure of the HIV PR dimer with an inhibitor bound in the active site. Monomers are colored blue and red, respectively. Regions involved in creation of a dimeric interface are highlighted by darker shades and indicated by residue numbers. **(b)** A detail of the four-stranded antiparallel β-sheet formed by interdigitation of C- and N-terminal strands. Monomers are colored blue and red, respectively. A hydrogen bonding network is represented by green dashed lines. The figure was generated using the structure of highly mutated, patient derived HIV-1 PR (PDB code 3GGU [8]) and program PyMol [9].

HIV PR is only active as a dimer in which each of the two catalytic aspartates is contributed by one monomer. The determination of the dimer dissociation constant (K_d_) has been the goal of considerable efforts of many groups, but its value differs substantially depending on the various techniques and experimental conditions (reviewed in [110]). The reported K_d_ values from kinetic studies are typically of the order of 10^−7^ to 10^−9^ M, whereas the values derived from sedimentation analysis are on average three orders of magnitude lower [111–117]. It was already suggested in 1990 that blocking the dimerization of the protease monomers could be an effective means for inactivating the enzyme [118]. The crystal structure of HIV-1 protease [119] shows that the enzyme dimerization interface is formed by the β-hairpin of the two flaps, catalytic triad, helices (residues 84–93) interacting with residues 4–10, and N- and C-terminal β-strands (Figure 8a). A four-stranded antiparallel β-sheet composed of the two N-termini (residues 1–4) interdigitating the two C-termini (residues 96–99) (Figure 8b) contributes to 75% of the stabilizing energy [120]. This dimeric interface is conserved among most HIV-1 isolates and drug-resistant variants (see Figure 2) and thus represents an attractive target for development of ligands preventing dimerization.

Early studies demonstrated that interface peptides reproducing the native sequence of C-terminal and N-terminal fragments act as HIV PR dimerization inhibitors, although in micromolar concentration [112,121,122]. Connection of the N- and C- terminal peptides with flexible linkers [123,124] or more rigid scaffolds (“**molecular tongs**”) [125,126] further increased their inhibitory potency. A recent study describes novel interfacial peptides tethered through their side chains, with inhibitory potencies against the wild-type HIV PR being in low nanomolar range [127].

Modification of the termini of an interfacial peptide by attachment of a lipophilic group and alkyl chains (e.g., palmitoyl) improves both the inhibitory capacity and the specificity [128,129]. Currently, the most potent lipopeptide inhibitors, containing the minimal peptide sequence Leu-Glu-Tyr modified on the N-terminus by palmitoyl, attained sub-nanomolar K_i_ values for the *in vitro* inhibition of the wild type and drug-resistant mutant variants [130]. Irreversible inhibitors, in which the interface peptide is able to covalently modify the protein dimeric interface through a disulfide bridge with Cys95, have also been described [131].

Interesting macromolecular inhibitors targeting dimerization domains are fusions of the N-terminal HIV-1 PR peptide with a cell permeable domain from HIV-1 Tat [132,133]. Also, an antibody recognizing the N terminus of HIV PR (residues 1–7), which inhibits activity of both HIV-1 and HIV-2 proteases with K_i_ values in low nano-molar range [134,135], has been reported. The use of the antibody as an anti-HIV drug is rather limited, nevertheless its structure [136,137] might be used as a lead in design of low-molecular mimics.

There has been a significant effort to develop dimerization inhibitors of HIV PR and characterize their binding on a structural level [138]. Also, there is great interest to develop inhibitors targeting protein-protein interfaces in enzymes and other proteins. Several protein–protein interfaces indeed became targets for successful drug development, e.g., HIV proteins and intracellular co-factors [139], HIV capsid assembly (reviewed in [140]) or herpes virus protease dimerization [141]. These advances might positively boost the development of new HIV PR dimerization inhibitors [142].

### 5.2. Inhibitors targeting HIV PR flaps

**Figure 9.**
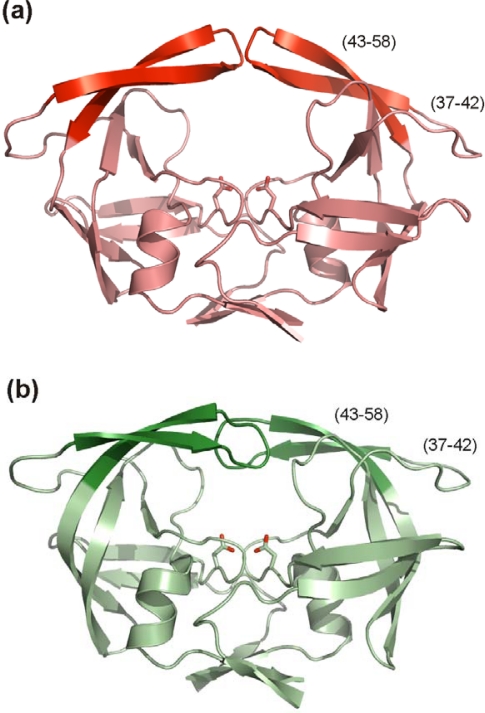
HIV PR flap conformations. **(a)** Overall structure of the apo-form of the HIV PR. The flaps (residues 43–58) in semi-open conformation are highlighted in red, residues 37–42, so called flap elbows are also indicated. The figure was generated using the structure of free HIV-1 PR (PDB code 1HHP [145]) and program PyMol [9]. **(b)** Overall structure of the HIV PR with flaps (in dark green) in closed conformation. Residues 37–42, so called flap elbows are also indicated. Inhibitor bound in the enzyme active site is omitted from the figure. The figure was generated using the structure of a highly mutated patient derived HIV-1 PR (PDB code 3GGU [8]) and program PyMol [9].

Residues 43–58 of HIV PR form an anti-parallel β-hairpin referred to as “the flap”. Two flaps within a dimer cover the active site cavity and their movement is crucial for binding and release of protease substrate. The conformation behavior of this glycine rich region has been extensively studied [143,144] and the most populated states are closed and semi-open. The closed state is found when a substrate or peptidomimetic inhibitor is bound to the active site (Figure 9b) while the semi-open conformation is most prevalent in the enzyme apo-form (Figure 9a). Since all currently approved FDA PIs target the closed conformation, developing of inhibitors targeted to the open flap conformation with a different binding mode might be an alternative to circumvent the cross-resistance.

Recent examples of inhibitors targeting the open-flap conformation are **metallacarborane**-based [101] and **pyrrolidine**-based [146] compounds. The metallacarboranes described earlier in this review bind to the flap-proximal part of the enzyme active site without interaction with the catalytic aspartates, while the pyrrolidine diester inhibitors target the catalytic dyad.

The region preceding the flap in the sequence (residues 34–42) is called the flap elbow (or the hinge). It folds into a surface exposed loop with no particular secondary structure which undergoes substantial change during flap opening and closing (Figure 9). With the exception of an insertion in position 35 [3], no resistance mutations are associated with this region (Figure 2, Table 1). The flap elbow thus might represent a promising drug target.

A monoclonal antibody recognizing an epitope corresponding to residues 36–46 inhibits the activity of HIV PR with K_i_ value in a nanomolar range [147]. The proposed inhibition mechanism based on the crystal structure of the antibody fragment in complex with the 36–46 epitope peptide postulates that antibody binding prevents flap closure over the active site [148]. An example of another inhibitory compound with predicted affinity toward the flap elbow are Nb-containing polyoxometalates [97], mentioned above. They inhibit HIV PR with K_i_ values in a low-nanomolar range and exhibit a non-competitive mode with stoichiometry 2 inhibitors to 1 PR dimer. Computational studies suggested that these compounds bind to a cationic pocket formed by residues Lys41, Lys43, and Lys55.

## 6. Conclusions

Development and clinical application of specific inhibitors of HIV PR almost immediately after the identification of HIV PR as a valid pharmaceutical target represents fascinating and, indeed, probably the most successful example of rational drug design in the history of biomedicine. There are currently nine FDA approved PIs available. In developed countries the protease inhibitors have at present time a secure position in the therapeutic armamentarium for both the initial therapy and the second-line and salvage treatment. It is also highly probable, that they will keep this position with further development of new more effective and less toxic molecules in the future [149]. Because of their high costs, protease inhibitors are in the resource-limited settings used mainly as drugs of the second-line therapy, but in the future an increase of their use can be certainly expected [150].

There is continuous need for the development of safer, cheaper drugs, active against the host of multi-resistant HIV species stemming from different virus strains, with high antiviral activity, excellent pharmacokinetic properties and little off-target activity, imposing low pill burden and little side-effect to the patient. Even if such an ideal drug never materializes, the research on HIV PR and its inhibition will continue to provide remarkable wealth of information about ligand-enzyme and protein-protein interactions, structural plasticity of proteins, mechanism of resistance development, pharmacokinetics of a chemotherapeutic in individual compartments of the body, *etc.* All this information could be used (and, indeed, is already being used) for the development of other compounds targeted against other pathogens, other enzymes and other, seemingly indomitable human diseases.

## Figures and Tables

**Table 1.** Mutations in the protease gene associated with resistance to PIs ^a^.

PI^b^	Major mutations^c^	Minor mutations^d^
Atazanavir +/− ritonavir	50, 84, 88	10, 16, 20, 24, 32, 33, 34, 36, 46, 48, 53, 54, 60, 62, 64, 71, 73, 82, 85, 90, 93
Darunavir^e^	50, 54, 76, 84	11, 32, 33, 47, 74, 89
Fosamprenavir^e^	50, 84	10, 32, 46, 47, 54, 73, 76, 82, 90
Indinavir^e^	46, 82, 84	10, 20, 24, 32, 36, 54, 71, 73, 76, 77, 90
Lopinavir^e^	32, 47, 82	10, 20, 24, 33, 46, 50, 53, 54, 63, 71, 73, 76, 84, 90
Nelfinavir	30, 90	10, 36, 46, 71, 77, 82, 84, 88
Saquinavir^e^	48, 90	10, 24, 54, 62, 71, 73, 77, 82, 84
Tipranavir^e^	33, 47, 58, 74, 82, 84	10, 13, 20, 35, 36, 43, 46, 54, 69, 83, 90

a Adapted from International AIDS society reports [7]

b Ritonavir is not listed separately as it is currently used only as a pharmacologic booster of other PIs (in low dose).

c Major mutations are those selected first in the presence of the drug or those substantially reducing drug susceptibility.

d Minor mutations emerge later and do not have a substantial effect on virus phenotype. They may improve replication capacity of viruses containing major mutations.

e PIs used in co-formulation with ritonavir.

**Table 2.** Overview of the inhibitors of HIV protease approved for clinical use with their dosage, side effects, and the position in the present therapeutic arsenal, elaborated with regard to the recommendations of the US Department of Health and Human Services [Panel on Antiretroviral Guidelines for Adults and Adolescents. Guidelines for the use of antiretroviral agents in HIV-1-Infected adults and adolescents. Department of Health and Human Services. November 3, 2008; 1–139. Available online: http://www.aidsinfo.nih.gov/ContentFiles/AdultandAdolescentGL.pdf. (Accessed 12 September 2009)]

**Generic name and abbreviation**	**Commonly recommended dosage**	**Most common side effects and off-target activities**	**Position in the present therapeutic arsenal**
**Ritonavir** (RTV)^a^	100–200 mg p.o. BID as pharmacokinetic booster of various PIs (original pharmacodynamic dose was 600 mg p.o.BID)	nausea, diarrhea, abdomenalgia, hyperlipidemia, lipodystrophy syndrome, inhibition of the cytochrome P450 3A4	practically only pharmacoenhancing of various PIs
**Saquinavir** (SQV)	1000 mg + RTV 100 mg p.o. BID	diarrhea, hyperlipidemia, lipodystrophy syndrome	second-line HAART therapy
**Indinavir** (IDV)	800 mg + RTV 100 mg p.o. BID	nephrolithiasis, lipodystrophy syndrome, hyperlipideamia, hepatotoxicity	second/third-line HAART therapy in case of resistance or intolerance
**Nelfinavir** (NFV)	1250 mg p.o. BID	diarrhea, hyperlipidemia, lipodystrophy syndrome	second/third-line HAART therapy in case of resistance or intolerance, approved for therapy of children
**Lopinavir** (LPV/r) ^b^	400 mg + RTV 100 mg p.o. BID	diarrhea, hyperlipideamia, lipodystrophy syndrome	first line option for PI based HAART regimen
**Amprenavir** (APV)	600 mg + RTV 100 mg p.o. BID	diarrhea, toxoallergic rash, hyperlipidemia, lipodystrophy syndrome	replaced by its prodrug fosamprenavir
**Fosamprenavir** (FPV)	700 mg p.o. + RTV 100 mg p.o. BID	diarrhea, toxoallergic rash, hyperlipidemia, lipodystrophy syndrome	first-line option for PI based HAART regimen
**Atazanavir** (ATV)	300 mg + RTV 100 mg p.o. q24h or 400 mg p.o. q24h	hyperbilirubinemia, ECG abnormalities (1° atrioventricular block)	first-line option for PI based HAART regimen
**Tipranavir** (TPV)	500 mg + RTV 200 mg p.o. BID	toxoallergic rash, hepatotoxicity, intracranial hemorrhage, lipodystrophy syndrome, diarrhea	second-line HAART therapy in case of resistance
**Darunavir** (DRV)	600 mg + RTV 100 mg p.o. BID or 800 mg + RTV 100 mg p.o. q24h	nausea, diarrhea, hyperlipidemia, headache, toxoallergic rash	recently approved for first-line HAART

Abbreviations: BID - twice per day, q24h - every 24 hours

a **Ritonavir** is at present used in therapy of HIV infection practically only as a pharmacokinetic booster.

b **Lopinavir** is marketed by the manufacturer only as (**Kaletra^®^**), in co-formulation together with low doses of ritonavir which acts as a pharmacokinetic booster.

## Abbreviations

APV: Amprenavir
ATV: Atazanavir
BCV: Brecanavir
BID: twice per day
CYP450 3A4: Cytochrome P450/3A4
DRV: Darunavir
FDA: Food and Drug Administration
FPV, fAPV: Fosamprenavir
HAART: Highly active antiretroviral therapy
IDV: Indinavir
K_d_: Dissociation constant
K_i_: Inhibition constant
LPV: Lopinavir
NFV: Nelfinavir
PIs: Protease inhibitors
PR: Protease
q24h: every 24 hours
RTV: Ritonavir
SQV: Saquinavir
TPV: Tipranavir
